# The hydrogen-bond collective dynamics in liquid methanol

**DOI:** 10.1038/srep39533

**Published:** 2016-12-20

**Authors:** Stefano Bellissima, Simone De Panfilis, Ubaldo Bafile, Alessandro Cunsolo, Miguel Angel González, Eleonora Guarini, Ferdinando Formisano

**Affiliations:** 1 Università di Firenze, Dipartimento di Fisica, Sesto Fiorentino, I-50019, Italy; 2 Istituto Italiano di Tecnologia, Center for Life Nanoscience, Roma, I-00161, Italy; 3 Consiglio Nazionale delle Ricerche, Istituto dei Sistemi Complessi, Sesto Fiorentino, I-50019, Italy; 4 Brookhaven National Laboratory, National Synchrotron Light Source II, Upton, New York 11973, USA; 5 Institut Laue-Langevin, Grenoble, F-38042, France; 6 Consiglio Nazionale delle Ricerche, Istituto Officina dei Materiali, Operative Group in Grenoble, F-38042, France

## Abstract

The relatively simple molecular structure of hydrogen-bonded (HB) systems is often belied by their exceptionally complex thermodynamic and microscopic behaviour. For this reason, after a thorough experimental, computational and theoretical scrutiny, the dynamics of molecules in HB systems still eludes a comprehensive understanding. Aiming at shedding some insight into this topic, we jointly used neutron Brillouin scattering and molecular dynamics simulations to probe the dynamics of a prototypical hydrogen-bonded alcohol, liquid methanol. The comparison with the most thoroughly investigated HB system, liquid water, pinpoints common behaviours of their THz microscopic dynamics, thereby providing additional information on the role of HB dynamics in these two systems. This study demonstrates that the dynamic behaviour of methanol is much richer than what so far known, and prompts us to establish striking analogies with the features of liquid and supercooled water. In particular, based on the strong differences between the structural properties of the two systems, our results suggest that the assignment of some dynamical properties to the tetrahedral character of water structure should be questioned. We finally highlight the similarities between the characteristic decay times of the time correlation function, as obtained from our data and the mean lifetime of hydrogen bond known in literature.

It is not common to find in Nature phenomena more intriguing than those related to the hydrogen bond interaction. This interaction dictates indeed not only the overall behaviour in organic molecules as DNA or proteins, but also that of systems as different as aqueous solutions and alcohols, which are undoubtedly the two classes of liquids having the larger impact on Life and daily Human activity and development, with innumerable applications in today’s life. The accurate knowledge of their properties is crucial to establish a firmer ground for the development of more refined liquid state theories, but it also has much broader societal implications. The full understanding of their microscopic behaviour is, however, far from being achieved even for the two most studied members of these families, namely water and methanol. This is mainly due to the presence of the hydrogen bond that induces strongly directional intermolecular interactions, absent in other non associated liquids. Comparing water to methanol at mesoscopic scales, as done in this paper, is rather natural and common. Methanol is indeed the alcohol having most analogies with water, their difference simply consisting in the replacement of a proton by a methyl group, that bestow on alcohols their amphiphilic character and different solvent properties. The continuous hydrogen bond (HB) breaking and formation is supposed on one hand to determine the overall behaviour of both liquids, and on the other to be at the root of their different structural, dynamical, and solvating properties. Liquid water, a system intensively studied in the search for a rationale for its unique properties and many unsolved anomalies^1^, has been long unanimously believed to have a dominating structure with a symmetric “ice-like” tetrahedral coordination assured by the two-donor and two-acceptor bonding scheme^2^,^3^,^4^. Completely different conformational properties are instead attributed to liquid methanol: although the “cyclic hexamer” structure originally conjectured by Pauling is, despite some controversy, still surviving, mixtures of long chains of variable lengths and/or rings of variable number of units are currently proposed to describe its liquid structure^5^,^6^.

However, the accepted and sharp structural distinction between these two liquids appears to fade according to the findings of recent experiments on water^7^,^8^. In these studies, a dominating combination of chains and rings, and the presence of thermally excited HB distorted structures were inferred, thus indirectly suggesting some analogy to the one-donor/acceptor methanol structure. However, also the last softer scenario has not escaped a strong criticism^9^,^10^,^11^, and is at the root of a vivid debate^8^,^12^,^13^,^14^. Recent simulations were used to reconcile these different views, suggesting that a strong and instantaneous energetic asymmetric HB distortion is able to explain the XAS results while keeping still valid the classical two donor/acceptor geometric HB picture^15^. HB interactions not only govern the stability and fluctuations occurring in these liquids, but also influence the dynamics over extended time scales (from fs to ps), and entail both diffusive and collective processes^16^. As to liquid methanol, molecular dynamics (MD) simulation results suggested a HB lifetime, *τ*_HB_, ranging from 1–2 ps^17^ to 5–7 ps^18^ at room temperature (RT), showing a difference which might depend on the intrinsic difficulties in defining the event of a HB breaking/forming when analysing a simulation. Slightly lower values, *τ*_HB_ ~ 1 ps, have been estimated for RT water by incoherent neutron scattering experiments^19^, but also by analysing simulations of SPC/E water^20^. Also, very recently, the numerical and THz laser spectroscopy investigation of ref. 21 provided evidence of the relation existing in the 1–10 THz range between collective longitudinal motions and the concerted coupling - mediated by the HB - of water tetrahedra, implying a complex interplay of different HB motions at ps time scales.

The investigation of the wavevector dependence of the THz excitations in liquid water, and their possible dispersive nature, can be readily performed by inelastic neutron (INS)^22^,^23^,^24^,^25^, x-ray scattering (IXS)^25^,^26^,^27^, and MD simulations^28^,^29^. Despite some controversies in the interpretation of the dispersion curves^30^, the coexistence of a low energy weakly dispersive mode and a high energy acoustic dispersive one is well established and observable also in slightly salty water^31^. The nature of the low frequency mode is more debated (see ref. 32 and references therein) and assigned to the coupling of density fluctuations with the bending of HB-linked O-O-O triplets^33^, which in ice was observed to have a mere transverse character^34^.

Unfortunately, THz spectroscopic methods couple at low *Q* primarily with longitudinal movements only, therefore not allowing us to directly examine the transverse character of a given mode. Consequently, any experimental signature of a shear wave propagation reflects a longitudinal-transverse (L-T) mixing^27^,^29^, that is the coupling between (acoustic) modes of orthogonal polarization. In the recent past, it has been conjectured that L-T coupling may appear in water due to its tetrahedral network structure^27^,^29^,^35^,^36^,^37^. Although often controversial, the information available on the collective dynamics of liquid water is much richer than the one on alcohols. Indeed, only a couple of INS^38^ and IXS^39^ experiments have been performed on liquid methanol, which, in addition, provided mutually inconsistent results, possibly owing to a limited energy resolution in the former case, or to the use of an oversimplified modelling in the latter. Furthermore, a recent joint quasi-elastic neutron scattering and MD work^40^ pinpointed an analogy between the structural relaxation in water and in methanol, although limitations in the dynamic range prevented this study from probing inelastic modes in the spectrum. The need of more accurate determination of the collective dynamics of liquid methanol and the possible similarities with water, prompted us to reconsider this still open, nearly unexplored, issue.

In order to perform a reliable characterization of the dynamical response of the sample, the synergy between numerical and experimental techniques is essential. Once the simulation results for the spectrum of density fluctuations are validated by the experimental ones, the dynamic structure factor *S(Q, E*) may then be computed and analysed in a much broader exchanged wavevector *Q* and energy *E* = *ħω* range, where *ħ* and *ω* represent the reduced Planck constant and the angular frequency, respectively. Within this approach, the neutron data are thus essential to benchmark, a posteriori, the ability of simulation, which in principle deals with a model system, to provide a realistic description of the dynamics in the actual system under study through the use of a reliable interaction potential.

## Results and Discussion

The study of the collective dynamics in amorphous systems and the determination of the dispersion law *ω(Q*) has often been performed using the neutron Brillouin scattering technique. This technique has to satisfy two main requirements. (i) Low scattering angles: because of long range disorder, collective phenomena show up indeed in these systems only at very low *Q*, in the so called pseudo first Brillouin zone which extends from the position of the main peak of the static structure factor down to the lowest possible *Q*, ideally to *Q* = 0. (ii) High energy incident neutron beam: since the collective excitations typically travel at velocities of the order of few thousands of m/s in liquids, the use of high energy (~10–100 meV) thermal neutrons is mandatory. Satisfying both conditions is extremely demanding at experimental level, because it implies the detection of high energy neutrons scattered from the sample at low angles: the contamination of the direct beam is unavoidable, and tight collimations must be used at the price of a strong flux decrease.

The neutron *BRI*llouin *SP*ectrometer BRISP^41^ is an instrument expressly built to fulfil the mentioned requirements, and has been therefore used to collect the experimental neutron Brillouin scattering spectra of liquid methanol at *T* = 298 K. Measured spectra were reduced, with standard procedures, to the intensity, *I*_exp_(*Q, E*) scattered by the sample, which is a quantity that contains, in addition to the sought-for single-scattering contribution, also a non negligible component due to multiple scattering (MS).

The MD simulations allow us to access a broader (*Q, E*) domain than in the experimental case and quantities that cannot be measured directly, such as: (i) the center-of-mass (CM) dynamic structure factor and (ii) the longitudinal and transverse currents^42^, these polarizations being defined, respectively as the direction parallel or orthogonal to the exchanged momentum *ħ**Q***.

**Figure 1:**
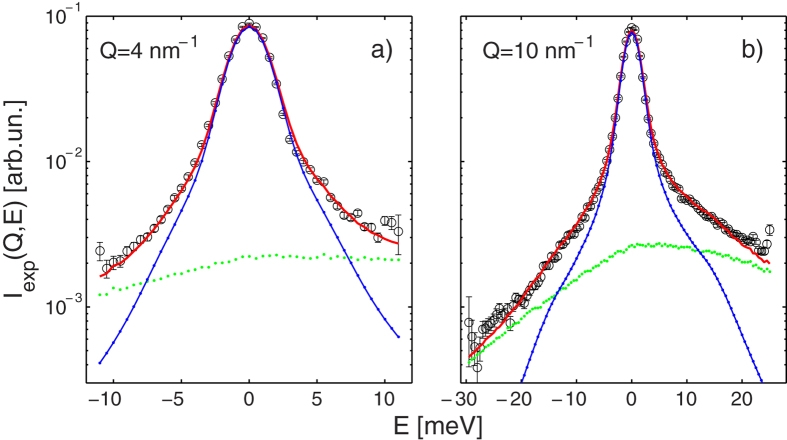
Fit of neutron Brillouin data obtained for liquid deuterated methanol at *T* = 298 K. The red line is the fit of a linear combination of the simulation (blue dots connected by a solid line) and multiple scattering (green dashes) data to the experimental spectra *I*_exp_(*Q, E*) (black circles with error bars) measured with a neutron incident wavelength of 1 Å; the *Q* values are displayed in the frames.

Figure 1 shows how the MD simulation data faithfully reproduce the experimental data *I*_exp_(*Q, E*) once the MS and instrumental resolution are taken into account, and emphasizes the overall accuracy of both measurements and calculations. More details on the experimental and calculated quantities can be found in the Sections *Methods* and *Supplementary Information*.

The examples shown in Fig. 1 highlight that the use of the OPLS-AA interaction model^43^ provides an excellent approximation of methanol spectra in a wide (*Q, E*) range, the more so if one realises that measurements with different neutron wavelengths and resolutions amount to independent determinations. This provides a solid ground to model the spectral response of the sample by the CM dynamic structure factor *S*_CM_(*Q, ω*) determined from the simulations. Complying with standard notation for the spectral variable, we now switch from the energy *E* to the angular frequency *ω*.

**Figure 2:**
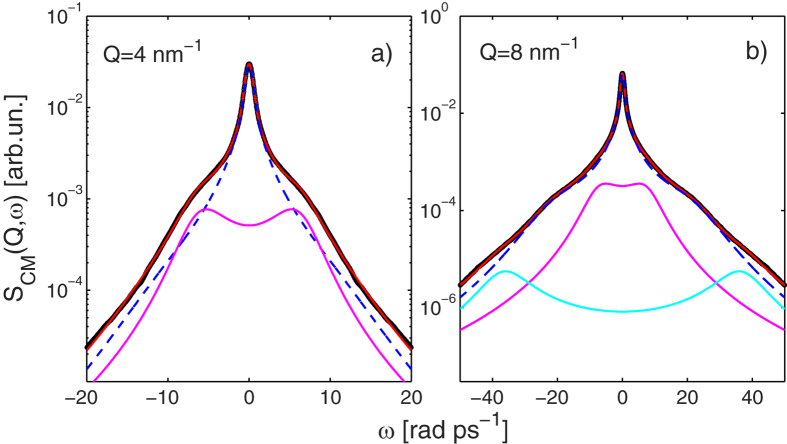
Fit of molecular dynamics results to the theoretical model for liquid deuterated methanol at *T* = 298 K. Fit of Eq. (1) to the simulated *S*_C*M*_(*Q, ω*) shown as black dots. The total fit (red solid line through the data points) is plotted together with the separate contributions of the VE (blue dashes), D1 (magenta line) and D2 (cyan) terms of the fit model. The *Q* values are indicated in the frames.

As in previous works on molecular fluids^44^,^45^,^46^, the *S*_CM_(*Q, ω*) is taken as the correct quantity representing the collective translational dynamics, which, however, is not unaffected by the molecular asymmetry and the anisotropy of the interaction, also due to the HB. The computed *S*_CM_(*Q, ω*) are plotted in Fig. 2. The logarithmic scale emphasises the presence of three very-low-intensity shoulders located at different frequencies, superimposed on the typical featureless decrease of the spectral wings. This behaviour is particularly evident at intermediate *Q* values. It is worth noticing that the highest-frequency mode, visible in the simulated spectra, was out of the experimental energy window and could not be covered by measured INS spectra.

Consistently with the long-debated case of water which, in recent experimental studies^25^,^30^, has been described by combining a standard viscoelastic (VE) dynamics model with the simplest expression able to give rise to another pair of inelastic lines, namely a damped harmonic oscillator (DHO), the function used to fit the *S*_CM_(*Q, ω*) reads:


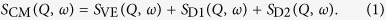


For the explicit definitions and detailed properties of the two models used in Eq. (1) we refer the reader to the *Methods* section and, for more detail, to refs 46 and 47. Here we only recall that the VE model gives a four-line spectrum, with two Lorentzian lines centred at *ω* = 0 describing the quasi-elastic response arising from the combined effects of thermal and viscous relaxations, while the frequency of the two other lines is related to the frequency *ω*_VE_ of the propagating sound wave. As to the DHO components, each of them contributes to *S*_CM_(*Q, ω*) through a further pair of Brillouin lines located at *ω*_D1,2_.

**Figure 3:**
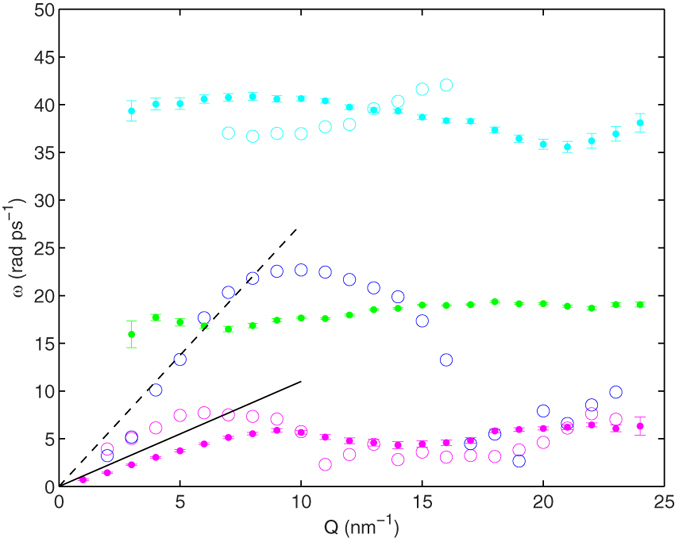
Energies of longitudinal and transverse modes for liquid deuterated methanol at *T* = 298 K. The frequencies obtained by modelling the *S*_C*M*_(*Q, ω*) of Eq. (1) are reported with open symbols and colours as in Fig. 2: *ω*_D1_(*Q*) (magenta), *ω*_V*E*_(*Q*) (blue), and *ω*_D2_(*Q*) (cyan), respectively); errors are within the size of symbols. The frequencies obtained from the analysis of the *J*_*T*_(*Q, ω*), namely *ω*_T*l*_(*Q*) (magenta), *ω*_T*i*_(*Q*) (green), *ω*_T*h*_(*Q*) (cyan), are represented with dots (see text and definitions preceding Fig. 4). The black line is the linear dispersion corresponding to the adiabatic sound speed of *c*_s_ = 1100 *m/s* while the dashed line corresponds to an apparent propagation velocity of ~2750 m/s, which has been determined by a linear fit of *ω*_V*E*_(*Q*) data up to 8 nm^−1^.

Examples of best fits of Eq. (1) to the simulated *S*_CM_(*Q, ω*) are shown in Fig. 2, where the logarithmic scale evidences an excellent fit quality over an intensity range of at least three orders of magnitude. The use of a model function with three pairs of inelastic lines clearly improves the fit of the simulated spectra in the 5–16 nm^−1^ *Q* range, while a model with only a DHO function was preferred at the lower or larger *Q*s. Best fit values of Eq. (1) yield the three dispersion relations *ω(Q*) reported in Fig. 3. The VE dispersion, *ω*_s_(*Q*), spans the intermediate energy values and exhibits the typical trend expected for a sound mode. The excitation propagates with an apparent propagation velocity ~2750 m/s, much larger than the adiabatic sound velocity (*c*_s_ = 1100 m/s), followed by the overdamping and bending down to a vanishing frequency around the position of the first maximum of the static structure factor, *Q*_p_ ≈ 17 nm^−1^. Conversely, both DHO components of *S*_CM_(*Q, ω*) display a softer mode not clearly dispersive, which remains underdamped in the whole *Q* range.

**Figure 4:**
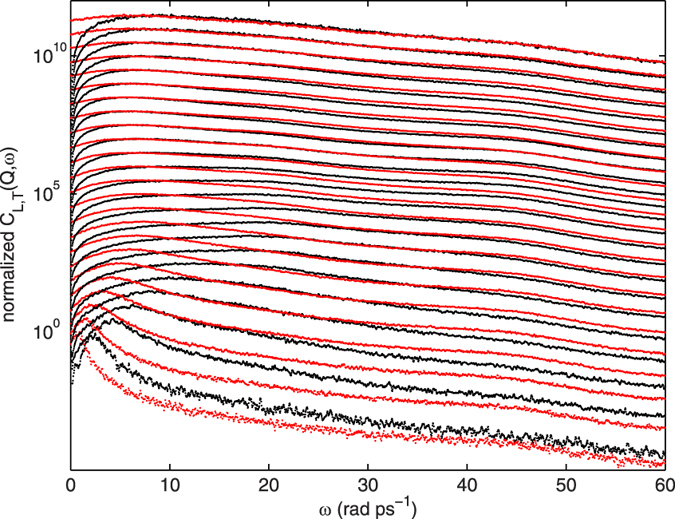
Longitudinal and transverse currents as determined by the molecular dynamics trajectories for liquid deuterated methanol at *T* = 298 K. The currents *J*_*L*_(*Q, ω*) (black) and *J*_*T*_(*Q, ω*) (red) are reported from *Q* = 1 to 24 nm^−1^ (from down to top) in a semi-logarthmic scale to emphasise the presence of the three modes; data are normalised to their respective maxima, and shifted for clarity.

Prior to a deeper discussion of these results, it is extremely useful to inspect the MD outputs, displayed in Fig. 4, for the longitudinal and transverse currents spectra, *J*_*L*_(*Q, ω*) and *J*_*T*_(*Q, ω*), respectively. The study of *J*_*L*_(*Q, ω*) = (*ω/Q)*^2^*S*_CM_(*Q, ω*) represents an alternative to the inspection of *S*_CM_(*Q, ω*), since it is a positive variable vanishing both at infinite and zero frequencies and, unless null everywhere, it must reach at least a maximum in between^42^. In the low *Q* limit, the position of the maximum tends towards the frequency of the dominant acoustic mode and it is customarily assumed to provide a reasonable estimate of this even at finite *Q* values. Conversely, although the *J*_*T*_(*Q, ω*) cannot be rigorously approximated by any known analytic function, the general features of its shape provide a meaningful characterisation of the transverse nature of the dynamics, and the frequency of the corresponding modes.

As evident from Fig. 4, the two currents display a clearly distinct behaviour at low *Q*; by increasing *Q*, we observe a gradual merging of the two spectra that become coincident at *Q* ≥ 15 nm^−1^, except for the intrinsic difference in the *ω* → 0 limit. The evidence of such progressive merging of the two polarisation states has been, to the best of our knowledge, never shown before, and it can be presumably seen as the effect on the currents of the transition from the continuum to the single particle regime: at low *Q*, when the system is probed over mesoscopic (few tens of Å) distances including many neighbours, the two polarisation states fully probe the anisotropic nature of the dynamics. Upon increasing *Q* and approaching, although still far by reaching, the single particle limit, this asymmetry gradually disappears together with the signs of any distinction between longitudinal and transverse dynamics.

Similarly to what has been observed for the *S*_CM_(*Q, ω*), both currents in Fig. 4 display three inelastic components. The current *J*_*T*_(*Q, ω*) clearly shows at all the *Q* investigated the presence of bumps related to excitations of transverse nature. The frequencies corresponding to these local *J*_*T*_(*Q, ω*) maxima, *ω*_Tl_(*Q*), *ω*_Ti_(*Q*), *ω*_Th_(*Q*), for the low, intermediate and high energy mode respectively, are compared to the frequencies obtained from the *S*_CM_(*Q, ω*) in Fig. 3; this comparison, though not completely rigorous, can be seen as the measure of the degree of the transverse nature in the truly dispersion mechanism contained in *S*_CM_(*Q, ω*).

Even more clearly than in Fig. 4, we note in Fig. 3 that the low energy transverse mode *ω*_Tl_(*Q*) has a slight, though evident, *Q* dependence, with a slope close to the methanol adiabatic sound velocity, but with the important difference that *ω*_Tl_(*Q*) does not show a positive dispersion as *ω*_s_(*Q*) does. Therefore, the analysis of *J*_*T*_(*Q, ω*) allows to attribute to the lower energy mode, *ω*_D1_(*Q*), a transverse and acoustic nature. The two transverse excitations at higher energy, in particular *ω*_Ti_(*Q*), are substantially *Q*-independent. Both are detectable at almost all *Q*s, and follow the typical behaviour of optic modes, as revealed by the non vanishing *Q* = 0 extrapolation of their characteristic frequency. The optic-like mode at *ω*_Ti_(*Q*) ~ 20 rad ps^−1^ is present in both *J*_*L*_(*Q, ω*) and *J*_*T*_(*Q, ω*) in Fig. 4; this mode was not detected in the analysis of the computed *S*_CM_(*Q, ω*) probably because the corresponding frequency was too close to that of the longitudinal acoustic mode, and therefore hard to separate from it. The values obtained for *ω*_Th_(*Q*) confirm the presence of a third mode at high energy, equivalent to *ω*_D2_(*Q*). Its persistence in a broad *Q* domain is clearly inferred when the currents are analysed.

Analogously to what has been observed for the current spectra of Fig. 4, we notice in the representation of Fig. 3 a merging of the acoustic (longitudinal) and low-frequency (transverse) dispersion curves at *Q* ≥ 15 nm^−1^. This seems consistent with the idea that being movements essentially localised and non-propagating, their dominant frequency becomes independent from both amplitude and direction of the exchanged momentum. This makes the mere concept of a mode polarisation ill-defined.

The ensemble of these findings reverses the interpretation of the collective dynamics of methanol. Due to their intrinsic weakness, the inelastic components of *S*_CM_(*Q, ω*) of methanol are even harder to detect in any spectroscopic method probing the total *S(Q, ω*) as often hidden by intramolecular dynamics. In x-ray spectroscopy, the difficulty is further complicated due to the typical long-tailed energy resolution function. In the IXS study of ref. 39 indeed, where no account was taken of these difficulties, *S(Q, ω*) spectra were analysed with a model fit function that intrinsically excludes the presence of two excitations and reduces to a simplified model composed of a single DHO supplemented by a single central Lorentzian line. While the neutron study of ref. 38 suffered from analogous limitations, the need of accessing *S*_CM_(*Q, ω*) through MD computations was correctly recognized in ref. 48, and its analysis led to the detection of excitations with energies in reasonable agreement with the two modes that we find at lower energies. However, a substantial difference is that we can now unambiguously identify, as well as firmly assign, a transverse character to the lowest frequency one.

Our findings suggest some similarities of the methanol dynamics with the long debated case of liquid water^24^,^25^,^27^,^30^. As previously reported for water, we observe a high frequency excitation mode with strong positive dispersion propagating at a velocity much higher than the adiabatic limit velocity. The velocity of this “fast” excitation is about 2750 m/s in methanol, which scales with the analogue quantity observed in water (3200 m/s) as the ratio between the respective *Q*_p_ values: this suggests a universal nature of dispersion in HB liquid dynamics^49^. The dispersive VE mode in methanol closely resembles a similar dispersive mode observed in liquid water (see ref. 25), except that the bending occurs at higher *Q*’s in water, probably owing to its larger *Q*_p_. In addition to this, we find the important result that methanol, as water, exhibits a second excitation at lower frequencies. This mode is only slightly dispersive but with a possible transverse acoustic origin that also in water has been suggested on the ground of a low *Q* linear dispersion with a slope smaller than, yet somewhat close to, the adiabatic sound velocity of water^35^. Like in water, the typical frequencies of this excitation span the 4–10 rad ps^−1^ range at *Q* ≥ 4 nm^−1^. This mode has been frequently related to a coupling between longitudinal and transverse dynamics arising in systems, like water, having a tetrahedral coordination^25^,^30^.

Even closer resemblances can be found in supercooled water, which shows a similar triple mode structure as reported in both an experimental^36^ and in a numerical study of TIP4P water^50^. A third high-energy and weakly dispersive mode, always emerging only at *Q* > 5 nm^−1^ as in methanol, but at higher energies, was also observed in the recent simulation of supercooled TIP4P/2005 water^51^. The authors of ref. 51 relate this behaviour to the tetrahedral structure in water and to the presence of a Boson peak analogously to what found in nanoconfined water^32^,^52^. However, given the lack of any tetrahedral coordination in liquid methanol, our results cast serious doubts on any interpretation of the water dynamic features in terms of its tetrahedral structure, rather highlighting the role of the hydrogen bond network.

Analogies have been found also in the normal mode analysis of a few hydrogen bonded liquids^53^, which evidences a similar behaviour for HF, for which hydrogen-bond effects are even stronger than in water, while in ref. 54 mention is made of a second, non-dispersive, excitation explicitly attributed to the hydrogen bond dynamics, but having an energy higher than the acoustic one.

Based upon the results of ref. 53, the triple mode structure found from the analysis of the transverse current can be linked to the coupling of density fluctuations with modes involving triplets of hydrogen bonded methanol molecules. In particular, the results of such a work urge us to ascribe the low frequency mode to the bending of such triplets, while the intermediate and high *Q*-constant frequency modes are to be connected to the symmetric and asymmetric stretching of the HB connecting them. Therefore, in analogy with the picture proposed in ref. 50 for supercooled water, an essentially optical character can be assigned to the highest frequency mode of methanol observed here.

Moreover, we notice in Fig. 4 that the highly dispersive mode dominating *J*_*L*_(*Q, ω*) at low *Q*s rapidly vanishes being gradually taken over by a low frequency transverse mode at high *Q*s. A similar trend was previously observed by IXS measurements in water^27^, while it has no counterpart in non-associated fluids. The gradual emergence of weakly dispersing inelastic modes at short distances likely represents a not fully understood distinctive behaviour of HB systems and merits certainly further investigations. In conclusion, we here observed that methanol displays in the whole *Q* range a viscoelastic behaviour characterised by propagating sound modes which are increasingly damped with growing *Q*, until they become overdamped in a rather narrow range around the position of the main peak in the static structure factor *Q*_p_, where the sound propagation is arrested. This behaviour, already known to be a common property of a large variety of liquids^45^,^46^,^49^,^55^ described by exactly the same *S*_VE_(*Q, ω*), is also shared by methanol. In fact, if the acoustic mode dispersion *ω*_s_(*Q*) of methanol is compared to that of its non-hydrogen-bonded analog, i.e. methane, one sees that the two curves have substantially the same shape, differing only by a constant factor^45^. However, in contrast to methane, methanol shows also two additional (quasi-) non dispersive modes of translational origin.

**Figure 5:**
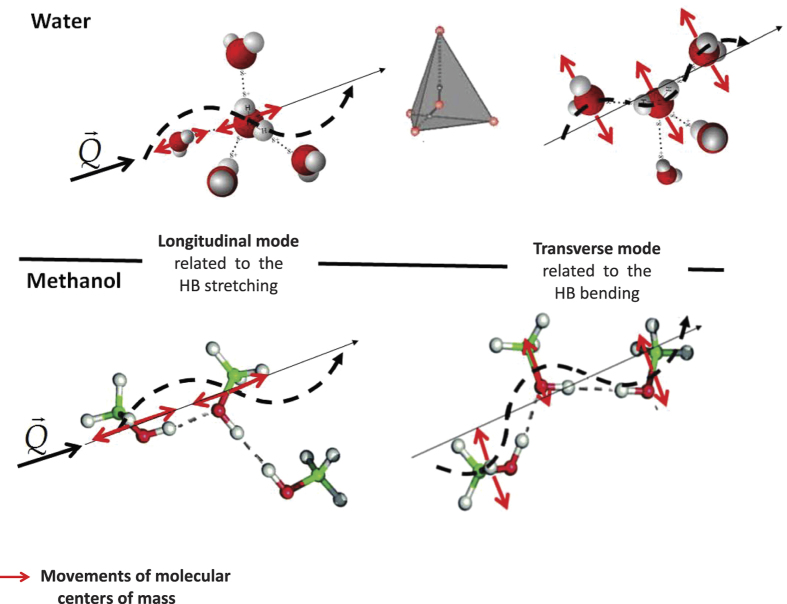
Schematic representation of longitudinal and transverse modes contributing to the THz spectrum of liquid water and methanol. The centers of mass movements of water and methanol’s molecules are respectively deduced from ref. 33 and from the analogies here observed between the THz dynamics of the two systems.

The main conclusion of our work is thus represented by the discovery of a methanol collective dynamics much richer and complex than known so far, and displaying features bearing evident similarities to those found in liquid water. Our data indirectly suggest that relevant dynamical features typically attributed to the tetrahedral coordination in water survive also in a non-tetrahedral liquid and it seems tempting to interpret the overall dynamics found for methanol as a sort of distinctive behaviour in HB systems. Figure 5 reports a pictorial representation of the coupling between the exchanged wavevector **Q** and the longitudinal and transverse waves propagating in water (tetrahedral) and methanol (non tetrahedral).

**Figure 6:**
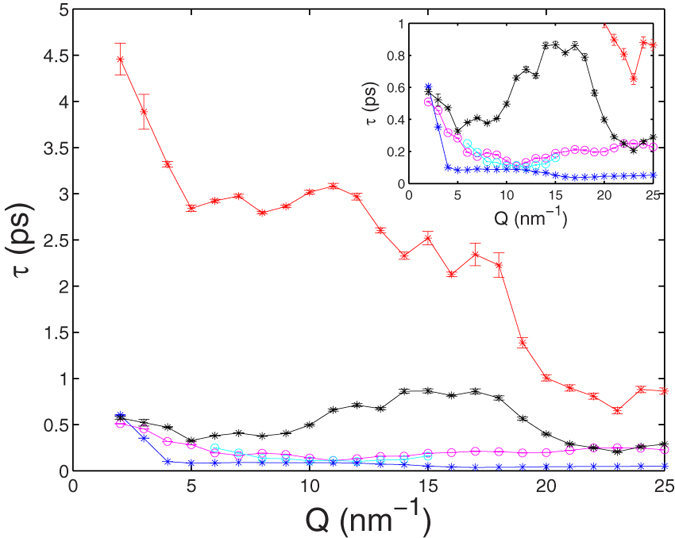
Characteristic lifetimes of the fitted model lines of liquid deuterated methanol at *T* = 298 K. Stars and open circles are VE and DHO lines, respectively. For the inelastic lines, the same colour code of Fig. 3 has been used. The lifetimes associated to quasi-elastic processes are shown in red (*τ*_1_) and black (*τ*_2_), respectively. The inset shows an enlarged view of the same data.

At a more speculative level, we may try to provide additional insight into the dynamics in methanol by recalling the strong asymmetry introduced by the HB, and assuming that its lifetime affects the decay time of all vibrational excitations implying HB bonds (such as e.g. the bending of O-O-O triplets). We recall indeed that our model is the frequency counterpart of time correlation functions having decay constants that reflect damping factors of both the VE and DHOs contributions in Eq. 1^47^,^56^. The five damping values associated to the three inelastic and two quasi-elastic excitations of the model of Eq. (1) give the corresponding five lifetimes *τ* reported in Fig. 6 (see *Methods*). The lifetimes corresponding to the three inelastic excitations shown in Fig. 3 are all in the ~0.1–0.3 ps range, while the two additional VE decay times of quasi-elastic origin are *τ*_1_ = 1/*z*_1_ ~ 1 ps, and *τ*_2_ = 1/*z*_2_ ~ 2 – 3 ps, respectively, at *Q* ≤ 15 nm^−1^.

All the *τ*’s reach an almost constant level at *Q* *~* 15 nm^−1^, which implies that localisation of all the vibrations occurs at the same *Q* where the longitudinal and transverse modes become almost undistinguishable. At *Q* ≤ 15 nm^−1^, we find that the two additional VE decay times, *τ*_1_ and *τ*_2_, have values similar to the HB mean lifetime obtained from simulations on methanol^17^,^18^. The existence of a direct link between the time parameters of the VE dynamics and the HB lifetimes is further confirmed by the strong decrease of *τ*_1_ and *τ*_2_ that we have found in parallel simulations ran with the methanol HB interaction turned off.

We finally remark that the dynamic scenario emerging from the present results seems compatible with the one observed in the Fast Infrared Spectroscopy study of the water OH stretching dynamics, where the coexistence of fast HB local oscillation (~200 fs) with a slower (~1 ps) correlation due to collective motions was proved^57^. It is then tempting to argue that in the case of HB systems, also the low-energy, and more typically intermolecular part of a dynamical response function may directly and quantitatively reflect the hydrogen bond dynamics, thus considerably reinforcing the picture of a strong entanglement between collective and localised modes.

## Methods

### The Brillouin scattering experiment

The small-angle spectrometer BRISP^58^,^59^ of the Institut Laue-Langevin (ILL, Grenoble, F) has been employed to record the Brillouin scattering of thermal neutrons from methanol at *T* = 298 K. A deuterated sample (CD_3_OD) was chosen in order to maximise coherent scattering and the visibility of collective dynamics. The measurements were carried out with two values of the incident-neutron wavelength (*λ*_0_ = 1 and 2 Å) for a more efficient coverage of the (*Q, E*) region while keeping the neutron speed sufficiently larger than the adiabatic sound speed in methanol (*c*_s_ = 1100 m/s^60^) and, at the same time, ensuring an adequate energy resolution function. The latter has a Gaussian shape, *G*_exp_(*E*) with a measured full width at half maximum of 3.0 and 0.7 meV for the two neutron wavelengths. The scattering angle range (1° ≤ *θ* ≤ 14°) allowed by the BRISP setup made possible the exploration of dynamical excitations at wave vectors between *Q* = 2 and 16 nm^−1^, the upper bound being close to the position of the main peak in the static structure factor *Q*_p_ = 17 nm^−1^.

#### Molecular dynamics simulations

MD simulations have been performed using the DL POLY 2.20 package^61^ to model a system of 4000 methanol molecules interacting through the OPLS-AA intermolecular potential^43^. Intramolecular bond distances were constrained to the equilibrium values (r_*CH*_ = 1.09 Å, r_*CO*_ = 1.41 Å, r_*OH*_ = 0.945 Å) by means of the SHAKE algorithm, while the remaining intramolecular motions such as bond-angle bending and rotation of the methyl group around the axis of the C-O bond were treated using a harmonic and a cosine potential, respectively, with the corresponding OPLS-AA parameters which are reported in the *Supplementary Information*. This model has proven to be able to reproduce in a satisfactory way the density and heat of vaporization of liquid methanol^62^, as well as its structure^63^. The equations of motion were integrated using the leapfrog algorithm with a timestep of 1 fs. Lennard-Jones interactions were truncated using a cutoff of 12 Å, while the Coulomb interactions were treated using the method of Ewald sums, with the same cutoff of 12 Å for the real space part. A random initial configuration was equilibrated during 500 ps in the NPT ensemble at 298 K and 1 bar using Berendsen’s thermostat and barostat with a coupling constant of 1 ps for both temperature and pressure. Then the system was equilibrated for another 500 ps using the NVT ensemble with Berendsen’s thermostat and finally a simulation of 100 ps was performed under the same conditions (NVT ensemble, Berendsen’s thermostat, coupling constant 1 ps) and during this run instantaneous atomic positions and velocities were saved every 10 steps (i.e. 0.01 ps). These were then used to compute the center-of-mass trajectory allowing to compute the dynamic structure factor and the longitudinal and transverse currents in a broad range of wavevectors 1 < *Q* < 24 nm^−1^, and *ω* up to ~350 rad ps^−1^.

It is also worth noting that MD simulations have also been carried out with the H1 potential in ref. 17 where rigid molecules are assumed. As the use of these data in Eq. (1) provided results of lesser accuracy, we have not discussed them in this paper.

#### The model

The VE contribution in Eq. (1) contains (i) two inelastic terms proportional to


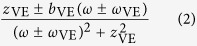


where the parameter *b*_VE_ makes the inelastic Lorentzian lines asymmetric with respect to frequency of sound excitations *ω*_VE_ with a damping *z*_VE_, and (ii) two Lorentzian quasi-elastic terms proportional to


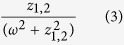


with damping *z*_1,2_^47^.

The DHO model is expressed by a stripped-down version of the same spectral profile, where only the two inelastic terms


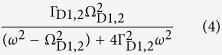


survive, Ω_D1,2_ and Γ_D1,2_ being the frequency and damping of the excitations, respectively. The characteristic lifetime *τ*_1,2_ of a given excitation is simply obtained as the reciprocal of the respective damping.

## Additional Information

**How to cite this article**: Bellissima, S. *et al*. The hydrogen-bond collective dynamics in liquid methanol. *Sci. Rep.* **6**, 39533; doi: 10.1038/srep39533 (2016).

**Publisher's note:** Springer Nature remains neutral with regard to jurisdictional claims in published maps and institutional affiliations.

## Supplementary Material

Supplementary Information
