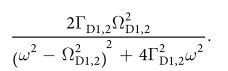# Corrigendum: The hydrogen-bond collective dynamics in liquid methanol

**DOI:** 10.1038/srep43809

**Published:** 2017-03-14

**Authors:** Stefano Bellissima, Simone De Panfilis, Ubaldo Bafile, Alessandro Cunsolo, Miguel Angel González, Eleonora Guarini, Ferdinando Formisano

Scientific Reports 6: Article number: 3953310.1038/srep39533; published online: 12 20 2016; updated: 03 14 2017

This Article contains typographical errors in Equation (4), where


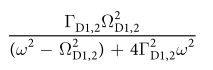


should read