# Supplementary material for: The hydrogen-bond collective dynamics in liquid methanol
Source: Sci Rep. 2016 Dec 20;6:39533. doi: 10.1038/srep39533 (PMC5172242; doi:10.1038/srep39533)
Supplement: Supplementary Information [file srep39533-s1.pdf]

The hydrogen-bond collective dynamics in liquid methanol.

Supplementary information.

Stefano Bellissima[1], Simone De Panfilis[2], Ubaldo Bafile[3], Alessandro Cunsolo[4],

Miguel Angel González[5], Eleonora Guarini[1], and Ferdinando Formisano[6]

[1] *Dipartimento di Fisica, Università di Firenze, Italy*

[2] *CLNS, Istituto Italiano di Tecnologia, Roma, Italy*

[3] *CNR-ISC, Sesto Fiorentino, Italy*

[4] *National Synchrotron Light Source, NSLS II, Brookhaven National Laboratory, New York, USA*

[5] *Institut Laue Langevin, Grenoble, France and*

[6] *CNR-IOM, Operative Group in Grenoble, France*

VALIDATION OF EXPERIMENTAL AND SIMULATION DATA

Once corrected the raw data for background noise, container scattering, and sample self-attenuation, we have checked the reliability of the simulations in reproducing the experimental data $I_{\rm exp}(Q,E)$. To this purpose, the latter have been fitted with a linear combination of the simulated dynamic structure factor $\tilde{S}_{\rm sim}(Q,E)$ and the multiple scattering contribution $I_{\rm MS}(Q,E)$, evaluated by the method described in Ref. 1.

We have performed, at each Q, linear least-squares-fits of

$$\begin{aligned} I_{\rm exp}(Q,E) = C\tilde{S}_{\rm sim}(Q,E) \otimes G'(E) + \\ + K I_{\rm MS}(Q,E) \otimes G_{\rm exp}(E) \end{aligned} \tag{1}$$

to the data corrected for background contribution, yet still including multiple scattering. The only two fit parameters C and K are normalization factors, that turned out to be Q-independent, as expected. The function $\tilde{S}_{\rm sim}(Q,E)$ denotes the simulated neutron-weighted dynamic structure factor, which contains the scattering lengths of the various atomic species according to the neutron scattering double-differential cross section of molecular systems.

The $\tilde{S}_{\rm sim}(Q,E)$ profile, already multiplied by a factor to account for the detailed balance, is convoluted with a function $G'(E)$ such that $G'(E) \otimes G_{\rm sim}(E) = G_{\rm exp}(E)$, where $G_{\rm sim}(E)$ describes the intrinsic broadening of simulated spectra. It has to be noted that $\tilde{S}_{\rm sim}(Q,E)$ and $I_{\rm MS}(Q,E)$ are obtained from independent calculations. After this check, we have safely calculated the $S_{\rm CM}(Q,\omega)$, with $\hbar = E/\omega$, being $\hbar$ the reduced Planck constant and E the energy exchanged between the neutron and the sample.

The $S_{\rm CM}(Q,\omega)$ is a quantity which cannot be extracted from experimental data unless one resorts to approximations concerning the separability of translational and rotovibrational motions and the degree of anisotropy of the intermolecular potential. Such approximations are inaccurate in molecular fluids, unless they present highly symmetrical interactions and molecular structures, which is surely not the case of methanol.

PARAMETERS OF THE INTRAMOLECULAR POTENTIAL

The parameters of the OPLS-AA potential [2] used to model the methanol dynamics are reported below.

**Atom**	σ[**Å**]	ϵ[**kJ/mol**]	**q**[**e**]
C (CT)	3.5	0.276144	+0.145
H methyl (HC)	2.5	0.125520	+0.040
O (OH)	3.12	0.711280	-0.683
H hydroxil (HO)	0	0	+0.418

**Bending**	$\mathbf{K_b}$[**kJ/mol/deg^2**]	θ_0[**deg**]
HC-CT-HC	276.144	107.8
HC-CT-OH	292.880	109.5
CT-OH-HO	460.240	108.5

**Torsion**	$\mathbf{V_3}$[**kJ/mol**]
HC-CT-OH-HO	1.8828

SUPPLEMENTARY TABLE S1. The Lennard-Jones parameters, partial charges, force constants and equilibrium angles of the OPLS-AA model for methanol [2]. The Lennard-Jones interactions between different atom types are obtained using the geometrical combination rules: $\sigma_{ij} = (\sigma_i \sigma_j)^{1/2}$ and $\epsilon_{ij} = (\epsilon_i \epsilon_j)^{1/2}$. The force constants and equilibrium angles are defined as $U(\theta_{ijk}) = 0.5 K_b (\theta_{ijk} - \theta_0)^2$, and $U(\Phi_{ijk}) = 0.5 V_3 (1 + cos(\Phi_{ijk}))$, for the bending and dihedral angle potential, respectively.

[1] De Francesco, A., Bafile, U., Formisano, F., and Guarini, E. (2012). Efficient implementation of multiple scattering Monte Carlo estimates in time-of-flight neutron spectrometry exploiting wide-area detectors. *Journal of Physics: Conference Series*, **340**(1), 012024.

[2] Jorgensen, W. L., Maxwell, D. S., and Tirado-Rives, J. (1996). Development and testing of the OPLS all-atom force field on conformational energetics and properties of organic liquids. *Journal of the American Chemical Society*, **118**(45), 11225–11236.
